# OP9 Feeder Cells Are Superior to M2-10B4 Cells for the Generation of Mature and Functional Natural Killer Cells from Umbilical Cord Hematopoietic Progenitors

**DOI:** 10.3389/fimmu.2017.00755

**Published:** 2017-06-30

**Authors:** Lara Herrera, Juan Manuel Salcedo, Silvia Santos, Miguel Ángel Vesga, Francisco Borrego, Cristina Eguizabal

**Affiliations:** ^1^Cell Therapy and Stem Cells Group, Basque Center for Transfusion and Human Tissues, Galdakao, Spain; ^2^Research Unit, Basque Center for Transfusion and Human Tissues, Galdakao, Spain; ^3^Immunopathology Group, BioCruces Health Research Institute, Barakaldo, Spain; ^4^Ikerbasque, Basque Foundation for Science, Bilbao, Spain

**Keywords:** hematopoietic stem cells, umbilical cord blood, natural killer cells, *in vitro* cell differentiation, immunotherapy

## Abstract

Adoptive natural killer (NK) cell therapy relies on the acquisition of large numbers of mature and functional NK cells. An option for future immunotherapy treatments is to use large amounts of NK cells derived and differentiated from umbilical cord blood (UCB) CD34^+^ hematopoietic stem cells (HSCs), mainly because UCB is one of the most accessible HSC sources. In our study, we compared the potential of two stromal cell lines, OP9 and M2-10B4, for *in vitro* generation of mature and functional CD56^+^ NK cells from UCB CD34^+^ HSC. We generated higher number of CD56^+^ NK cells in the presence of the OP9 cell line than when they were generated in the presence of M2-10B4 cells. Furthermore, higher frequency of CD56^+^ NK cells was achieved earlier when cultures were performed with the OP9 cells than with the M2-10B4 cells. Additionally, we studied in detail the maturation stages of CD56^+^ NK cells during the *in vitro* differentiation process. Our data show that by using both stromal cell lines, CD34^+^ HSC *in vitro* differentiated into the terminal stages 4–5 of maturation resembled the *in vivo* differentiation pattern of human NK cells. Higher frequencies of more mature NK cells were reached earlier by using OP9 cell line than M2-10B4 cells. Alternatively, we observed that our *in vitro* NK cells expressed similar levels of granzyme B and perforin, and there were no significant differences between cultures performed in the presence of OP9 cell line or M2-10B4 cell line. Likewise, degranulation and cytotoxic activity against K562 target cells were very similar in both culture conditions. The results presented here provide an optimal strategy to generate high numbers of mature and functional NK cells *in vitro*, and point toward the use of the OP9 stromal cell line to accelerate the culture procedure to obtain them. Furthermore, this method could establish the basis for the generation of mature NK cells ready for cancer immunotherapy.

## Introduction

Natural killer (NK) cells constitute 10–15% of peripheral blood (PB) lymphocytes and display a half-life of approximately 7–10 days in circulation (1). They can also be found in cord blood (CB) in a similar frequency to PB (2), but the small volume in CB units represents the difficulty in obtaining suitable numbers of NK cells needed for clinical use (3). Human NK cells are phenotypically described as CD3^−^CD56^+^ cells within the lymphocyte population (4), and they are classified as a subset within the group 1 of innate lymphocyte cells, capable of producing IFN-γ, and exert cytotoxicity (5). According to the intensity of the expression of the CD56 receptor, *in vivo* differentiated mature NK cells are divided into CD56^bright^ and CD56^dim^ subpopulations (6). CD56^bright^ cells constitute less than 10% of circulating NK cells, produce high levels of inflammatory cytokines, and have none or low expression of CD16. CD56^dim^ NK cells express CD16 and contain an abundance of granules that arm them for cytolytic activity against viral-infected and cancer cells (7). NK cells are originated from CD34^+^ hematopoietic progenitors (4). Before reaching a mature stage, they acquire progressively and orderly different surface markers, being classified into stage 1 (CD34^+^, CD45RA^+^, CD117^−^, CD94^−^, CD56^−^, CD16^−^), stage 2 (CD34^+^, CD45RA^+^, CD117^+^, CD94^−^, CD56^−^, CD16^−^), and stage 3 (CD34^−^ CD117^+^, CD94^−^, CD56^−^, CD16^−^). Once they reach a mature stage, NK cells are phenotypically described by their surface markers as stage 4 (CD34^−^, CD94^+^, CD117^+/−^, CD56^bright^, CD16^+/−^) and stage 5 (CD34^−^, CD94^+/−^, CD117^−^, CD56^dim^, CD16^+^) (8).

Current NK cell-based cancer immunotherapy aims to reverse the tumor-induced NK cell dysfunction that is observed in patients with cancer and to increase and sustain NK cell effector functions (9, 10). The low numbers of these cells in PB and, even lower numbers in CB, have led to several approaches to expand and/or activate freshly isolated autologous or allogeneic NK cells by culturing with different interleukins, such as IL-2, IL-15, and IL-21 (11–14). CD34^+^ hematopoietic progenitors from umbilical cord blood (UCB) are being considered a source for the production of a large number of NK cells (15, 16). Obtaining NK cells from UCB CD34^+^ hematopoietic progenitors has been extensively described (17). However, further research is needed to obtain even larger numbers of mature and functional NK cells ready to use in cancer immunotherapy.

In this study, we aimed to evaluate the production of functional and mature NK cells from UCB CD34^+^ hematopoietic progenitors with two different culture conditions, where OP9 and M2-10B4 cell lines are used as feeder layers. OP9 is typically used as a support for the differentiation of CD34^+^ cells from embryonic stem cells (ESCs) or pluripotent stem cells (18–21). Instead, M2-10B4 is a good support to maintain CD34^+^ cells in a long-term culture, acting like a hematopoietic niche (22). Our data show that these two culture conditions generated a large number of mature and functional NK cells. Furthermore, the presence of OP9 feeder cells in the culture generated a higher amount of mature NK cells in a faster manner when compared with culture conditions with M2-10B4 feeder cells.

## Materials and Methods

### Umbilical Cord and PB Samples and Cell Lines

Umbilical cord blood and PB samples were obtained with prior signed informed consent and ethical committee approval from the Basque Ethics Committee for Clinical Research [Comité Etico de Investigación Clinica de Euskadi-CEIC-E (PI2014138)]. Fully signed written informed consent was obtained from the pregnant mothers. UCB units that contain between 1.5 × 10^9^ and 8 × 10^8^ mononuclear cells were used for investigation purposes. One fresh UCB unit (less than 30 h between the extraction and the processing) was used to perform a set of experiments (Table S1 in Supplementary Material). OP9, M2-10B4, and K562 cell lines were purchased from ATCC (CRL-2749, CRL-1972, and CCL-243, respectively). OP9 cells were cultured with α-MEM (Gibco), 20% fetal bovine serum (FBS) (Hyclone), 1% penicillin/streptomycin, and 1% Glutamax. M2-10B4 cells were culture with RPMI, 10% FBS (Hyclone), 1% penicillin/streptomycin, and 1% Glutamax. Finally, K562 cells were cultured with RPMI, 10% FBS (Hyclone), 1% penicillin/streptomycin, 1% Glutamax, 1% NEAA, and 1% sodium pyruvate.

### Hematopoietic Stem Cell (HSC) Differentiation Protocol into NK Cells

Umbilical cord blood mononuclear cells were obtained by density gradient using Ficoll-Paque™ PLUS (GE Healthcare). Then, HSCs were isolated by MACS sorting, using the CD34 MicroBead kit from Miltenyi Biotec. CD34^+^ cells (5,000 cells/well) were plated onto 6-well plates coated with OP9 or M2-10B4 cells inactivated with Mitomycin C (10 µg/ml) (Sigma) and plated in feeder-free system and cultured with the media described by Ni et al. (23): Ham F12^+^ DMEM (1:2), 20% human serum (AB serum-Invitrogen, Life Technologies), 1% penicillin/streptomycin, 2-mercaptoethanol (25 µM), ascorbic acid (20 μg/ml), and sodium selenite (5 ng/ml). At the beginning of the differentiation process, IL-3 (5 ng/ml), IL-7 (20 ng/ml), IL-15 (10 ng/ml), SCF (20 ng/ml), and FLT3 ligand (10 ng/ml) (Miltenyi Biotec) were added to the medium. Half of the medium was changed every week. From the second week of differentiation, IL-3 was no longer added to the medium as was described by Cichocki and Miller (24) and Grzywacz et al. (25). The differentiation protocol that we carried out consisted of plating purified CD34^+^ cells over two different culture conditions using two feeder cells layers, OP9 and M2-10B4, and cultured for 42 days with the differentiation medium previously described. From day 14 up to day 42 of differentiation, immunophenotype analyses were performed, along with cytotoxicity and degranulation assays at 28, 35, and 42 days of differentiation.

### Flow Cytometry Analysis

Purity of CD34^+^ sorted cells from UCB samples was analyzed with CD34-PE antibody (BD Biosciences, clone 581) in a FACS Canto II (BD Biosciences). Purity of the CD34^+^ cells isolated had to be higher than 80% in order to perform our protocol of differentiation (Figure S1A in Supplementary Material). The number of remaining CD56^+^ cells in the purified sample was not significant (Figure S1B in Supplementary Material).

Different populations and maturation stages of *in vitro* differentiated NK cells were analyzed by flow cytometry at 14, 21, 28, 35, and 42 days in culture. Cells were washed with PBS/10% FBS and incubated for 30 min at 4°C for labeling with anti-CD94-FITC (BD Biosciences, clone HP-3D9), anti-CD117-PE (Miltenyi Biotec, clone A3C6E2), anti-CD56-APC (Biolegend, clone MEM-188), and anti-CD16-BV421 (BD Biosciences, clone 3G8). Next, cells were fixed and permeabilized with BD Cytofix/Cytoperm™ Plus in order to label them with anti-Perforin-PerCP-eF710 (BD Biosciences, clone δG9) and anti-Granzyme B-BV510 (BD Biosciences, clone GB11). 50,000–100,000 events were acquired for analyses. Populations were analyzed using FlowJo v.X.0.7 (TreeStar Inc.).

### Cytotoxicity Assay

In order to check the *in vitro* lytic activity of the differentiated NK cell against the K562 target cell line, we performed a calcein-AM-based cytotoxicity assay (26). K562 cell line was used as target cells. 10^6^ cells were incubated for 30 min at 37°C with 15 µM of calcein-AM (Life technologies C3099). These cells were washed twice after incubation. Calcein-AM-labeled K562 cells were cocultured with NK cells differentiated from CD34^+^ progenitors from UCB in a U-bottom 96-well plate for 4 h at 37°C at different ratios (25:1, 12.5:1, 6.25:1, and 3.125:1). As a control, we used NK cells from adult healthy donors’ blood isolated with the NK Cell Isolation Kit from Miltenyi Biotec (catalog number 130-092-657). These adult PB-NK cells were stimulated overnight under the same conditions as our UCB CD34^+^ *in vitro* differentiation protocol (IL-7, IL-15, SCF, and FLT3). Adult PB-NK cells were purified by MACS sorting, using the NK Cell Isolation Kit from Miltenyi Biotec (130-092-657). For measurement of spontaneous release, K562 target cells were incubated with no NK cells. Total released was achieved by adding 4% Triton™ X-100 (Sigma-Aldrich) to the target cells. Each condition was performed in triplicates. After the incubation, 100 µl of supernatant was collected and transferred to a black 96-well plate to measure the calcein-AM release in a Fluoroskan Ascent (Thermo Fisher) (excitation filter: 485 ± 9 nm; band-pass filter: 530 ± 9 nm). The percentage of specific lysis is calculated according to the following formula: [(Test release) − (Medium fluorescence)] − [(Spontaneous release) − (Medium fluorescence)]/[(Total release) − (Triton fluorescence)] − [(Spontaneous release) − (Medium fluorescence)] × 100.

### Degranulation Assay

Natural killer cells were cocultured with K562 target cells at ratio 1:1 in a 24-well plate for 6 h at 37°C. At the beginning of the assay, anti-CD107a BV421 (BD Biosciences, clone H4A3) was added in order to detect the degranulation activity of the effector cells against the target cells. Golgi Stop™ (BD Biosciences) (monensin) was added following the manufacturer’s protocol. After the incubation, cells were collected, washed, and labeled with anti-CD94-FITC and anti-CD56-APC. Degranulating NK cells (CD107a^+^) were determined in the CD56^+^ cells, both on stage 3 (CD56^+^CD94^−^) and stages 4–5 (CD56^+^CD94^+^) cells.

### Data Analysis

Differences between groups were evaluated using paired Student’s *t*-test. *p*-Values <0.05 were considered significant. Statistical calculations were done using GraphPad Prism 6 (GraphPad Software, Inc.) Bars represent the mean and error bars represent the SEM.

## Results

### OP9 Cell-Based Coculture System Generates Higher Numbers of CD56^+^ NK Cells than M2-10B4 Cell-Based Coculture System

**Figure 1:**
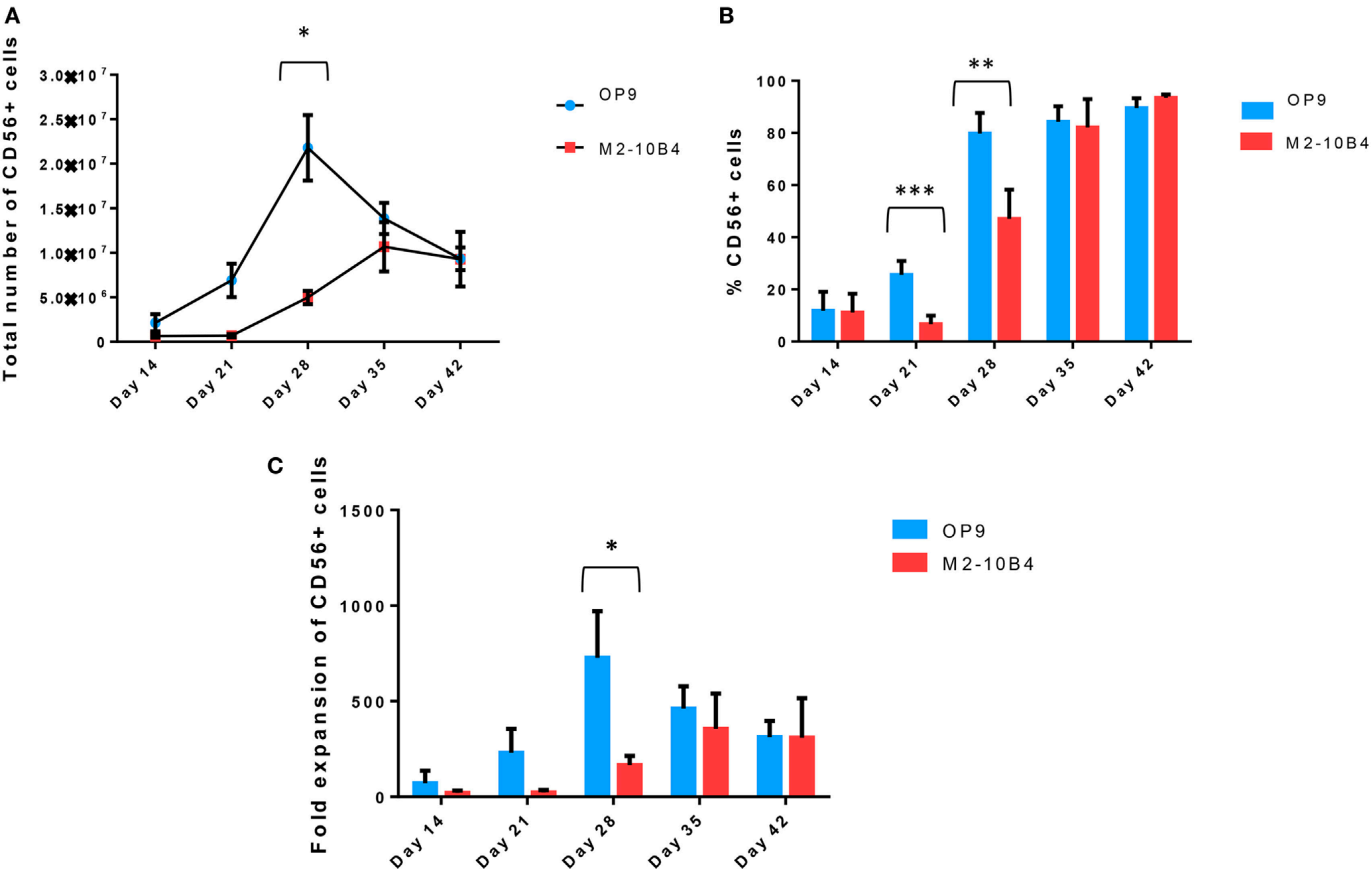
**(A)** Total number of CD56^+^ cells at different time points during the *in vitro* differentiation protocol with the two different culture conditions: OP9 and M2-10B4 cells coculture. **(B)** Percentage of CD56^+^ cells at different time points of the differentiation protocol with the two different culture conditions: OP9 and M2-10B4 cells coculture. **(C)** Fold expansion of the CD56^+^ cells regarding the initial number of CD34^+^ cells plated in each experiment (*n* = 4). The bars represent the mean and error bars represent SEM. *p*-Value: *p < 0.05, **p < 0.005, and ***p < 0.001. The absence of any asterisk indication means there are no significant differences.

CD34^+^ UCB cells were cultured up to 42 days using the protocol described in Section “Materials and Methods.” Cell number and percentage of CD56^+^ NK cells were checked weekly. CD34^+^ UCB cocultured with OP9 cells feeder layer exhibited a better proliferative capacity as compared with CD34^+^ UCB cocultured with M2-10B4 cells feeder layer at day 21. In addition, we found a higher number of CD56^+^ NK cells with OP9 cells coculture at 28 days of differentiation (*p* < 0.05), reaching 2 × 10^7^ NK cells on average, while this number dropped in the next days. Meanwhile, the number of CD56^+^ cells in the M2-10B4 cells coculture condition increased gradually until 35 days of differentiation, reaching 1 × 10^7^ NK cells, and dropped slightly at 42 days of differentiation (Figure 1A). Similarly, we observed a higher frequency of CD56^+^ cells in the OP9 cells coculture than in the M2-10B4 cells coculture, with significant differences at day 21 (*p* < 0.001) and day 28 (*p* < 0.01) (Figure 1B). Likewise, we observed a higher fold expansion of CD56^+^ cells in the OP9 cells coculture condition, especially at day 28 of differentiation (*p* < 0.05) which correlates with the pattern followed by the number of CD56^+^ cells obtained (Figure 1C). Also, we perform a feeder-free culture condition. Our results showed that a very small number of NK cells were obtained in this culture condition (Figure S2 in Supplementary Material). We believe that the presence of the feeder layer is crucial for the correct differentiation of NK cells from CD34^+^ cells.

### The *In Vitro* Differentiation Pattern of NK Cells Resembles the *In Vivo* Differentiation Pattern

According to the differentiation pattern from CD34^+^ UCB to mature CD56^+^ CD3^−^ NK cells *in vivo* (Figures S3 and S4 in Supplementary Material), key markers were selected in order to determine the *in vitro* differentiation pattern obtained under our differentiation protocols (27). Depending on the presence or absence of different markers, NK cells were classified in different stages (*Stage* < *3*: CD56^−^, CD94^−^, CD117^low^; *Stage 3*: CD56^+^, CD94^−^, CD16^−^, CD117^high^; *Stage 4*: CD56^+^, CD94^+^, CD16^−^, CD117^low^, and *Stage 5*: CD56^+^, CD94^+^, CD16^+^, CD117^low^), which were determined according to the expression of the NK cell markers CD56 and CD94. The correct analysis of the stages was assured by the expression of the CD117 marker on NK cells. CD56^+^/CD94^+^ population was divided in stages 4 and 5 based on the presence or absence of the CD16 marker. In this manuscript, stages 4 and 5 were represented as stages 4–5 in order to include mature NK cells in a single population.

**Figure 2:**
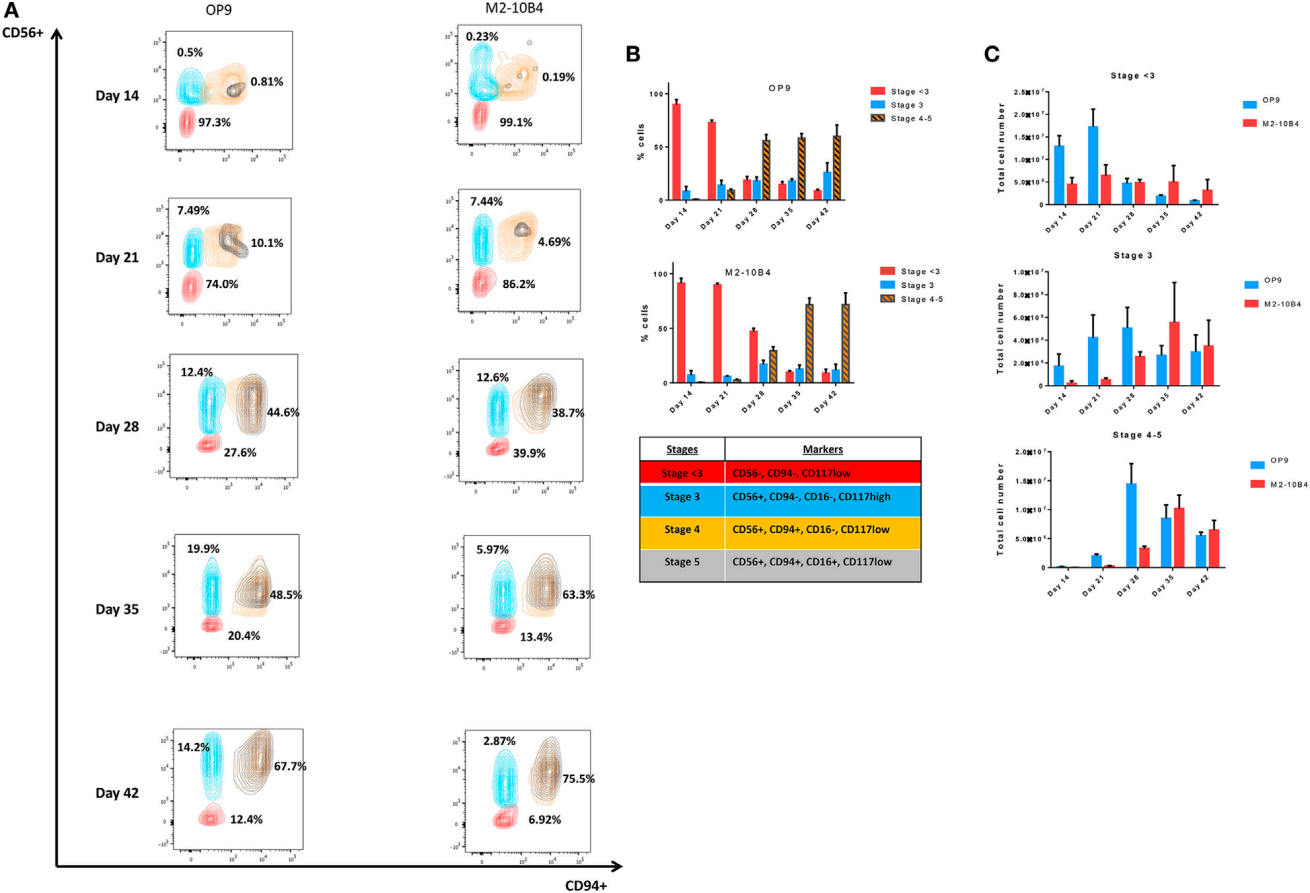
**(A)** Contour plots representing the different natural killer (NK) cell maturation stages during the differentiation protocol using OP9 and M2-10B4 cells as feeder layers. CD94, CD56, and CD16 markers were analyzed to determine the maturation stage as described in the text. Numbers represent the percentage of cells in stage <3 (red), stage 3 (turquoise), and stages 4–5 (orange and black). **(B)** Percentage of NK cells in the different stages of maturation (<3, 3, and 4–5) along the differentiation time course. **(C)** Total number of cells in the different stages (<3, 3, and 4–5) cultured with the two conditions: OP9 and M2-10B4 cells coculture. The bars represent the mean and error bars represent SEM (*Stage* <*3*: CD56^−^, CD94^−^, CD117^low^; *Stage 3*: CD56^+^, CD94^−^, CD16^−^, CD117^high^; *Stage 4*: CD56^+^, CD94^+^, CD16^−^, CD117^low^, and *Stage 5*: CD56^+^, CD94^+^, CD16^+^, CD117^low^).

The percentage of more mature NK cells increased over time during the *in vitro* differentiation protocol (Figure 2A), with higher percentage of more mature stages in culture conditions in the presence of OP9 cells in comparison with cultures in the presence of M2-10B4 cells. On the one hand, these more mature stages are reached earlier by the cells cocultured with the OP9 cell line than with the M2-10B4 cell line and the percentage of cells in the stages 4–5 is maintained over time with the OP9 cell line, while it gets higher with the M2-10B4 (Figure 2B). On the other hand, the total number of cells at stages 4–5 of maturation equalizes between the two culture conditions at the end of the differentiation protocol, having more cells at day 28 with the culture condition using OP9 cells (Figure 2C).

### *In Vitro*-Generated NK Cells Exhibit Cytotoxic Activity

**Figure 3:**
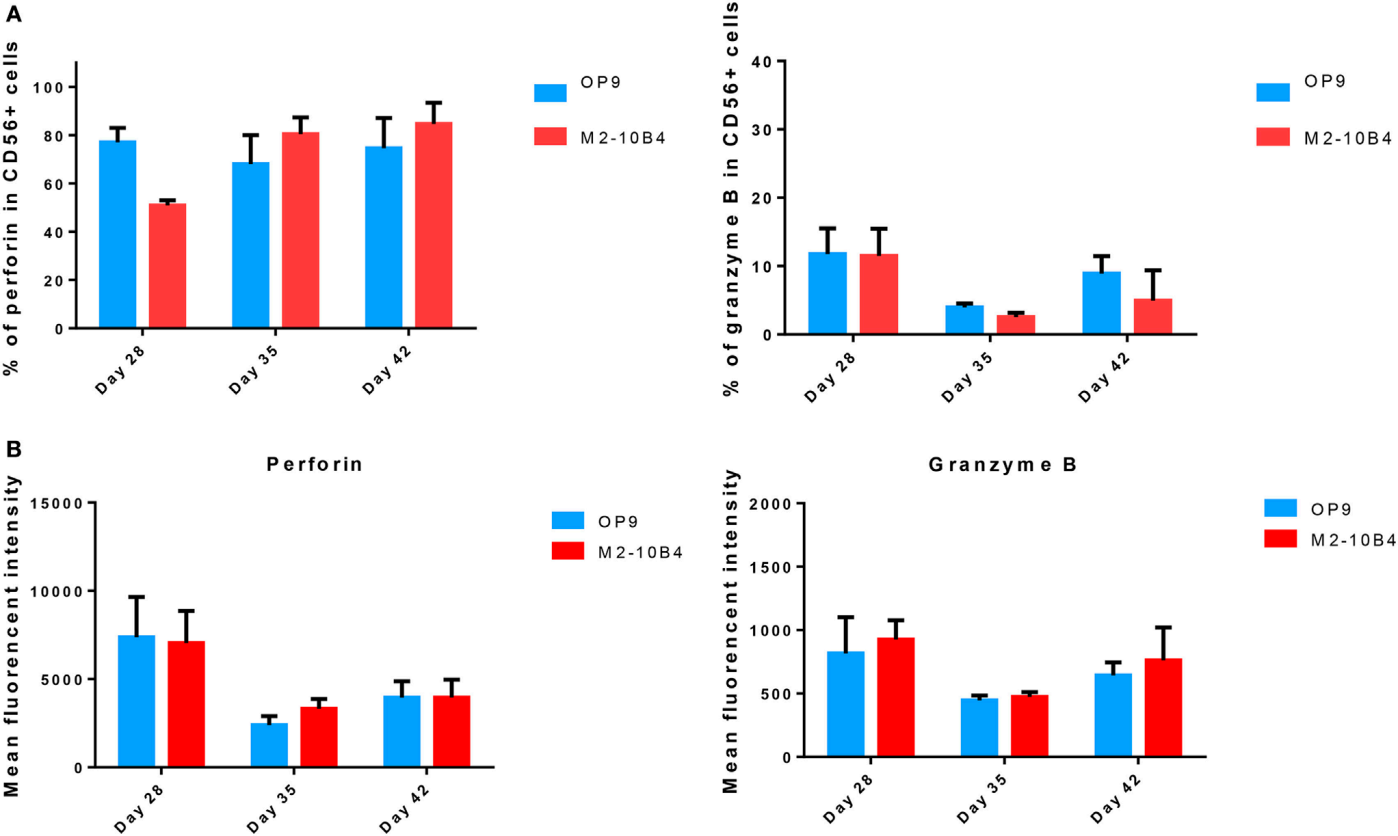
**(A)** Percentage of CD56^+^ natural killer (NK) cells expressing perforin and granzyme B cultured with OP9 and M2-10B4 feeder cells at days 28, 35, and 42 of the differentiation protocol. **(B)** Mean fluorescence intensity of perforin and granzyme B of CD56^+^ NK cells generated in the presence of OP9 and M2-10B4 feeder cells at days 28, 35, and 42 of differentiation. *p*-Value: *p < 0.05, **p < 0.005, and ***p < 0.001. The absence of any asterisk indication means there are no significant differences.

Mature NK cells (stages 4 and 5) express cytolytic granules containing perforin and granzyme (28). We determined the expression of perforin and granzyme B in developing NK cells at days 28, 35, and 42 of the differentiation protocol. No significant differences in the expression of these two cytolytic markers were found between cells cultured with the OP9 cells layer in comparison with cells cultured with the M2-10B4 feeder cells. In both culture conditions, the percentage and the intensity of perforin expression in positive cells was higher than the expression of granzyme-B (Figures 3A,B). Moreover, the frequency of cells expressing perforin at days 28, 35, and 42 with OP9 cells feeder layer was very similar to the percentage of CD56^+^ NK cells, as well as to the condition with M2-10B4 cells feeder layer at days 35 and 42. We also found that *in vitro* differentiated NK cells did not show a significant difference in the expression of perforin and granzyme B between stage 3 and stages 4–5 in both culture conditions (data not shown).

**Figure 4:**
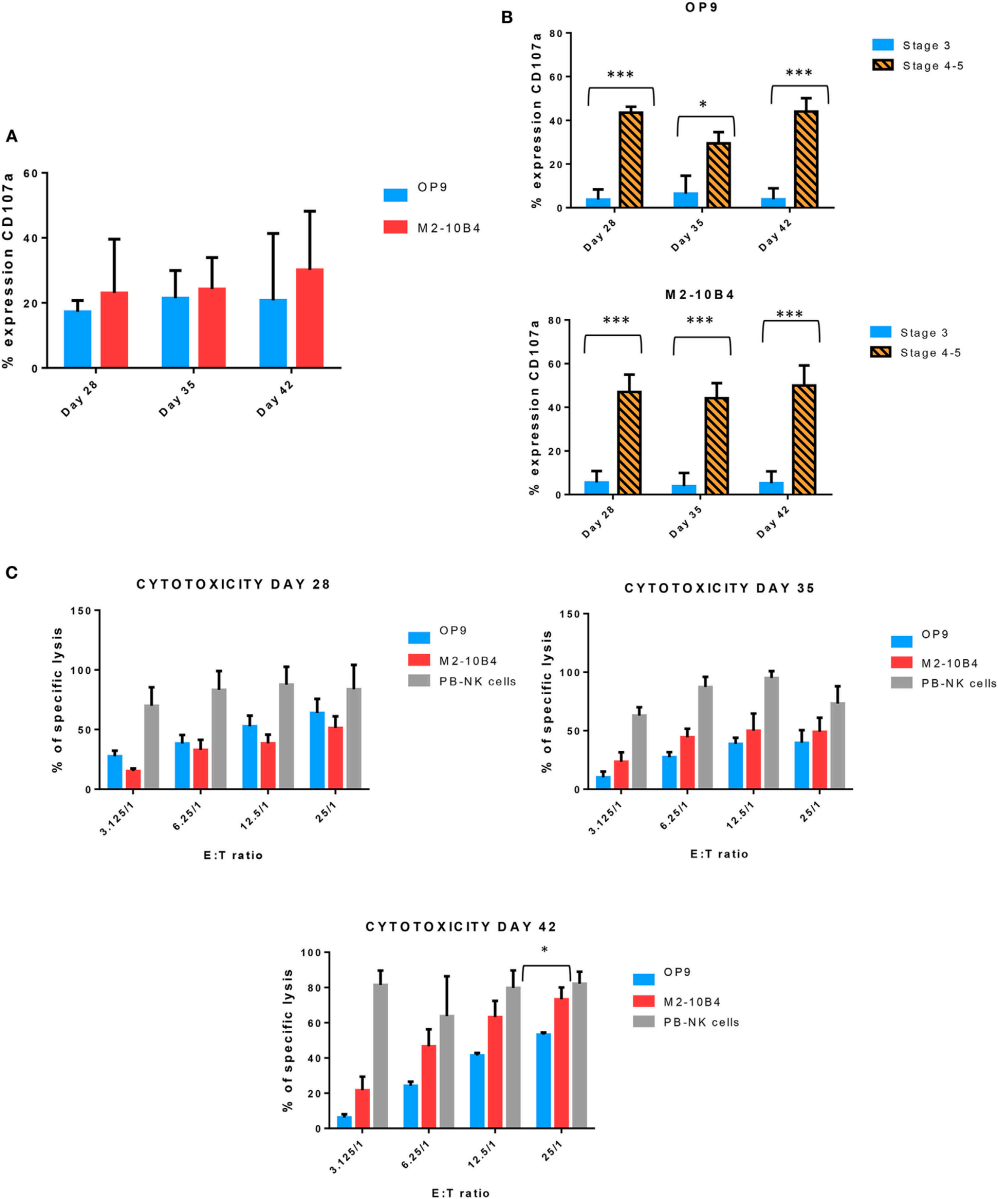
**(A)** Expression of CD107a, a marker of degranulation, in the *in vitro*-generated natural killer (NK) cells (CD56^+^) at different time points of the differentiation protocol in response to the stimulation with K562 target cells. **(B)** Expression of CD107a in both conditions (with OP9 and M2-10B4 feeder cell layers) at stage 3 and stages 4–5 at different times of the differentiation protocol. **(C)** Cytotoxicity activity of NK cells against K562 target cells at days 28, 35, and 42 of the differentiation protocol. Overnight cultured NK cells obtained from adult peripheral blood stimulated with the same cytokines as our *in vitro*-generated NK cells were used as a control. The bars represent the mean and error bars represent SEM. *p*-Value: *p < 0.05; ***p < 0.001. The absence of any asterisk indication means there are no significant differences (*Stage* <*3*: CD56^−^, CD94^−^, CD117^low^; *Stage 3*: CD56^+^, CD94^−^, CD16^−^, CD117^high^; *Stage 4*: CD56^+^, CD94^+^, CD16^−^, CD117^low^, and *Stage 5*: CD56^+^, CD94^+^, CD16^+^, CD117^low^).

Next, we determined the degranulation capacity of the *in vitro*-generated NK cells. To do that, we measured the expression of CD107a on NK cells after being stimulated with K562 target cells, according to the protocol described in Section “Materials and Methods.” Activated NK cells from PB of healthy adult donors were used as a control (data not shown). At days 28, 35, and 42, the degranulation tended to be higher in NK cells generated with M2-10B4 feeder cells than in the NK cells generated in the presence of OP9 feeder cells, although there were no significant differences (Figure 4A). We also analyzed the degranulation in the stage 3 and stages 4–5 of the *in vitro*-generated NK cells. The degranulation in both culture conditions exhibited a very significant difference between stage 3 and stages 4–5 (Figure 4B). Finally, we tested the cytolytic activity of NK cells against the K562 target cells. NK cells purified and activated from adult PB were used as a control. At day 28, NK cells differentiated in the presence of OP9 cells were more cytotoxic than NK cells differentiated in the presence of M2-10B4 cells (Figure 4C). At days 35 and 42, the cytotoxic activity was similar in both culture conditions at several effector:target ratios (Figure 4C). However, at day 42, at ratio 25:1, NK cells differentiated in the presence of M2-10B4 cells were significantly more cytotoxic than the NK cells differentiated in the presence of OP9 cells.

## Discussion

In this study, we tested and compared the effect of two different culture conditions during the generation of *in vitro* functional and mature NK cells from HSC precursors from UCB. We show that by using OP9 cells as a feeder layer we obtained higher number of CD56^+^ mature NK cells in comparison with M2-10B4 cells as a feeder layer. To date, several articles have described different protocols for *in vitro* NK cell differentiation from hematopoietic progenitors from UCB (CD34^+^ cells) including the usage of different feeder cells, cytokine cocktails, and time of culture (16, 17, 24, 25, 29–40). However, we believe that our study for the first time describes in detail the maturation stages of NK cells during the *in vitro* differentiation process in which a high number of functional NK cells are achieved with the possibility for using them in future immunotherapies against cancer.

We have described a new culture condition, using two cell lines as feeder layers, i.e., OP9 cells and M2-10B4 cells, to generate NK cells from UCB CD34^+^ hematopoietic precursors.

Other authors have described the use of these two cell lines to differentiate CD34^+^ cells from pluripotent stem cells, such as ESCs and induced pluripotent stem cells (12, 18, 19, 21) and also to support and maintain CD34^+^ cells in a long-term culture (41, 42).

The use of feeder cell lines with the aim of maintenance and differentiation of stem cells toward blood lineage cells is an usual practice. First, the cell line AFT024 was described by Moore et al., immortalized with SV-40 T antigen, and derived from murine fetal liver stromal cells (43). Specifically, to study human NK cell ontogeny, Miller and McCullar suggested that NK cell differentiation from CD34^+^ cells and receptor acquisition was contact dependent with the feeder layer AFT024 (44). Other groups investigated the AFT024 and EL08-1D2 potential to generate *in vitro* NK cells and found that EL08-1D2 is significantly better at recapitulating NK cell development (39).

M2-10B4 is the other feeder cell line commonly used, and it derives from murine bone marrow stromal cells. M2-10B4 has been used earlier for NK cell expansion (45, 46) and others determined its differentiation potential for NK cell generation from hESCs (47). Remarkably, these findings suggest the need of stromal cell microenvironment, and the importance of direct contact with the feeder layer.

Finally, few reports have used the OP9 cell line (38, 40) to differentiate NK cells from bone marrow CD34^+^ cells. Also, OP9-DL1 (OP9 modified with Notch ligand delta-like 1) is used to develop T lymphocytes (48) and NK cells from hematopoietic precursors (49). To summarize, the majority of published data indicate the need of a microenvironment supported by stromal cells. This microenvironment provides necessary factors for the correct maturation of NK cells. Therefore, we wanted to explore new culture conditions to obtain high number of mature and functional NK cells from UCB CD34^+^ cells, to improve the *in vitro* differentiation protocols previously published, and also to in detail characterize there *in vitro* development in comparison with the maturation stages described *in vivo*.

To do this, we have compared both cell lines (OP9 and M2-10B4) in terms of differentiation capacity, number of CD56^+^ cells and NK fold expansion from the first week up to 6 weeks of differentiation. In general, we have obtained higher number, fold expansion, and frequencies of CD56^+^ NK cells, specifically when they are generated in the presence of OP9 feeder cells, than other authors who have used similar protocols. For example, with the EL08.1D2 cell line as feeder cells, several groups have obtained a similar fold of expansion (16) or a lower number and frequency of CD56^+^ NK cells (25, 31, 39) than us. Others have also obtained lower number or lower fold expansion when the AFT024 cell line (39) or Stro-1^+^ cell line (32) were used as feeder layers. In our study, using both cell lines (OP9 and M2-10B4), we get a higher number of NK cells with OP9 cell line. Importantly, we have also obtained higher number and frequencies of CD56^+^ NK cells than others who have also used OP9 cells as feeder layer (38, 40).

Currently, the vast majority of researchers accept a linear model of differentiation of human NK cells with five stages of maturation, each characterized by a pattern of expression of surface receptors, functional capabilities, and differentiation potential (27). In stage 3, cells have variable expression of the markers CD161 and CD56, typical of mature NK cells, but they do not express inhibitory receptors for MHC class I molecules, i.e., KIR and CD94/NKG2A, which they are characteristic of mature NK cells. In addition, cells in stage 3 have the two signs of functional identity of mature NK cells: IFN-γ production and perforin-dependent cytotoxic activity. CD94 expression marks the transition to stage 4 in the development of human NK cells. Cells at this stage are characterized by high CD56 expression. The acquisition of CD16 in some CD94^+^ cells is considered a marker of cells in stage 5, the group defined by the phenotype CD94^high^CD56^dim^CD16^+^ cells. Therefore, CD56^dim^ and CD56^bright^ cells in PB represent the two terminal stages of differentiation of human NK cells (50).

We have observed that our *in vitro* differentiation and maturation process of NK cells follows a similar pattern regarding the surface markers acquisition, which are observed *in vivo*. For example, CD117 expression in our *in vitro* NK cells is always present in late stages (4, 5), whereas is downregulated in *in vivo* differentiated NK cells at stage 5. We believe this is due to the presence of SCF in our culture system, as it has been reported that the presence of this cytokine upregulates the expression of CD117 in CD56^+^ NK cells and significantly increases the capacity of CD56^bright^ NK cells to degranulate (51). In our cultures, we cannot distinguish between CD56^dim^ and CD56^bright^ NK cells, which probably are due to the fact that this *in vitro* phenotype is slightly different to the *in vivo* due to the culture conditions.

We observed differences in maturation stages between the two cell lines used as feeder layers. More mature stages are reached earlier by using OP9 cell line than with the M2-10B4 cell line. Otherwise, the total number of cells at stages 4–5 of maturation equalizes between the two conditions at the end of the differentiation protocol, having more NK cells at day 28 when the culture conditions include the OP9 feeder cells, which also may be responsible for the observed higher cytotoxicity activity. We think that these differences in timing of maturation and number is due to the NK cell differentiation potential properties among stromal cell lines used in this study, because it has been reported that depending of the origin of the stromal cell lines used in hematopoietic differentiation, the features of maturation, and functionality of the terminal cell type could be different (52).

Few groups have described the *in vitro* developed NK cells in the presence of OP9 feeder cells. First, it has been reported that using OP9 cell line (38) during the *in vitro* development of NK cells from bone marrow or UCB CD34^+^ cells, the levels of TGF-β may influence the developmental progression and subset formation of NK cells, like CD56^bright^CD16^−^ subset, but there are no studies in the progression of NK stages *in vitro*. Second, other group (40) also using OP9 feeder cells, obtained 80% of CD56^+^ cells at 28 days, but they did not distinguish between different stages of maturation. In our case, we are able to achieve 70–80% of CD56^+^ cells at the same day of differentiation but we studied in detail that around 38–45% of CD56^+^ cells are already in stages 4–5, showing the typical markers of mature NK cells. Besides, other groups using other culture conditions achieved a minor percentage of CD56^+^ cells in stages more immature than we did (16, 36).

In general, we have also obtained better or similar results than others when we look at the expression of cytolytic markers, degranulation properties, and killing activity of *in vitro*-generated NK cells. For example, in feeder-free systems, *in vitro*-generated NK cells expressed lower levels of perforin (34, 36), lower (36) or equal (35) cytotoxic activity, and equal levels of granzyme B (36) and degranulation potential (29, 30). When NK cells were generated in the presence of the EL08.1D2, they exhibited similar levels of perforin and granzyme B (16), similar degranulation levels (16, 33), and lower cytotoxic activity (25). Finally, in a study where OP9 feeder cells were used for the differentiation of NK cells, the authors found that they expressed lower levels of perforin (40) compared with the NK cells we obtained in our study.

In conclusion, we found that the use of OP9 and M2-10B4 cell lines to generate NK cells *in vitro* from UCB CD34^+^ is a feasible option that offers the advantage of obtaining higher functional and mature NK cell numbers with enhanced killing capacity. To the best of our knowledge, this is the first and the most comprehensive study comparing these two culture conditions for the generation of NK cells from fresh UCB CD34^+^ cells, being OP9 cells culture condition better than M2-10B4 cells, highlighting the great potential for UCB CD34^+^ for future NK cell-based immunotherapy.

## Ethics Statement

This study was carried out in accordance with the recommendations and approval of “Basque Ethics Committee for Clinical Research [Comité Etico de Investigación Clinica de Euskadi-CEIC-E (PI2014138)]” with written informed consent from all subjects in accordance with the Declaration of Helsinki.

## Author Contributions

LH: collection and/or assembly of data, data analysis and interpretation, and manuscript writing. JS: collection and/or assembly of data and data analysis. SS: data analysis and interpretation. MV: final approval of manuscript and financial support. FB: conception and design, data analysis and interpretation, manuscript writing, and final approval of manuscript. CE: conception and design, collection and/or assembly of data, data analysis and interpretation, manuscript writing, and final approval of manuscript.

## Conflict of Interest Statement

The authors declare that the research was conducted in the absence of any commercial or financial relationships that could be construed as a potential conflict of interest.

## Supplementary Material

Table S1 Number of fresh umbilical cord blood (UCB) units, volume in milliliters, number of mononuclear cells per UCB, and CD34^+^ cells remaining after purification for each experiment. UCB were fresh, being not longer than 30 h after the extraction.

Figure S1 **(A)** Purity of CD34^+^ cell population from umbilical cord blood (UCB) obtained by immunomagnetic isolation. **(B)** Remaining percentage of CD56^+^ cells after purification of CD34^+^ cells from UCB.

Figure S2 Total number of CD56^+^ cells at different time points during the *in vitro* differentiation protocol with three different culture conditions: OP9 and M2-10B4 cells coculture (N:4) and feeder-free (N:2).

Figure S3 Key markers expressed at different stages through natural killer cell differentiation/maturation *in vivo* and *in vitro*.

Figure S4 Dot-plots of the expression of CD3 at different stages of natural killer cells during the differentiation protocol.
