# Supplementary material for: OP9 Feeder Cells Are Superior to M2-10B4 Cells for the Generation of Mature and Functional Natural Killer Cells from Umbilical Cord Hematopoietic Progenitors
Source: Front Immunol. 2017 Jun 30;8:755. doi: 10.3389/fimmu.2017.00755 (PMC5491543; doi:10.3389/fimmu.2017.00755)
Supplement: Table S1 — Number of fresh umbilical cord blood (UCB) units, volume in milliliters, number of mononuclear cells per UCB, and CD34+ cells remaining after purification for each experiment. UCB were fresh, being not longer than 30 h after the extraction. [file Table_1.PDF]

Experiment №	UCB units	Volume (ml)	Mononuclear cells ($x10^6$)	CD34+ cells after purification
1	1	105.0	1136.1	325000
2	1	111.4	1366.878	315000
3	1	111.4	1340.142	350000
4	1	144.5	1473.9	287000
