# Supplementary figures and images for: OP9 Feeder Cells Are Superior to M2-10B4 Cells for the Generation of Mature and Functional Natural Killer Cells from Umbilical Cord Hematopoietic Progenitors
Source: Front Immunol. 2017 Jun 30;8:755. doi: 10.3389/fimmu.2017.00755 (PMC5491543; doi:10.3389/fimmu.2017.00755)

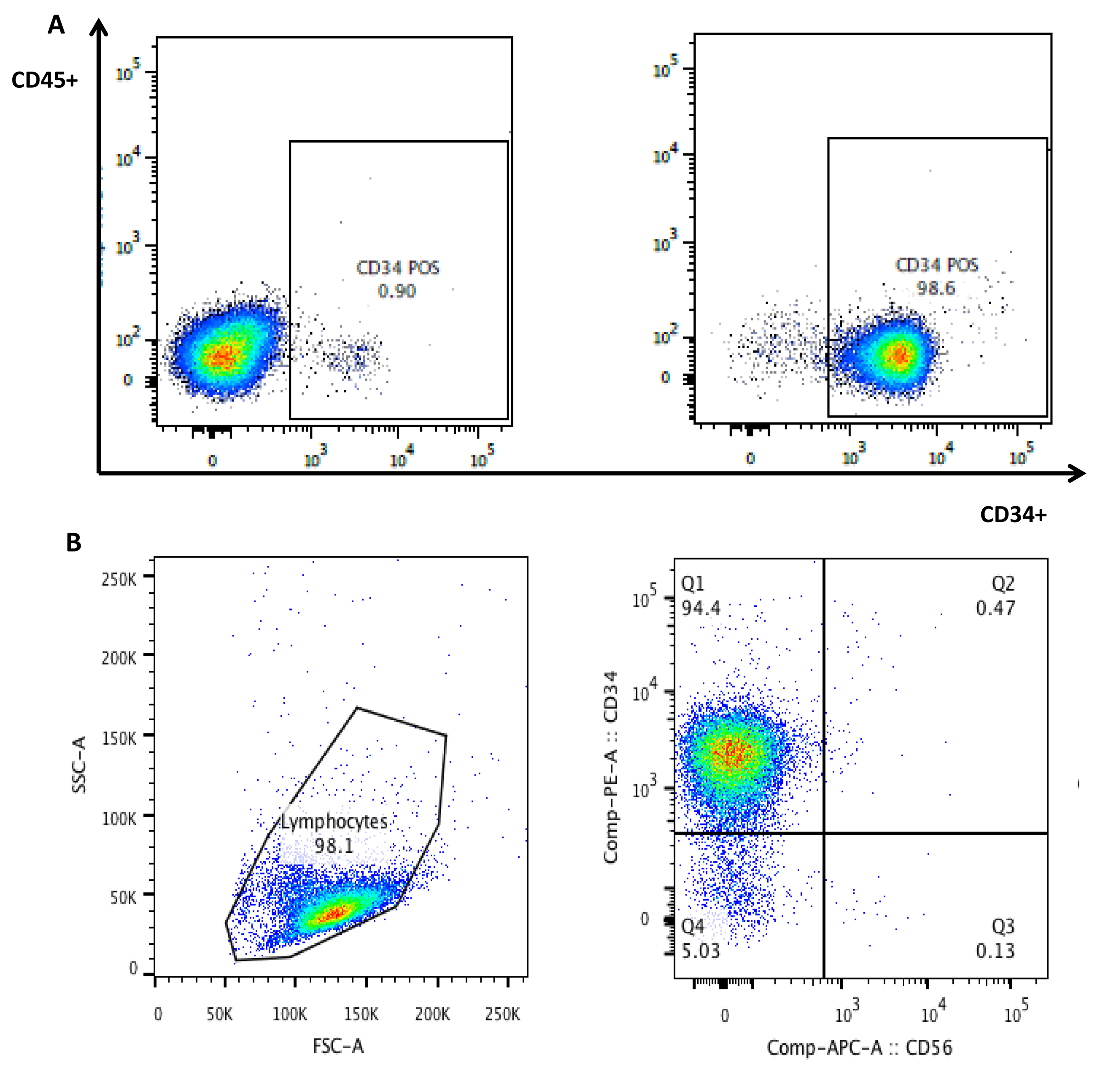

Supplement: Figure S1 — (A) Purity of CD34+ cell population from umbilical cord blood (UCB) obtained by immunomagnetic isolation. (B) Remaining percentage of CD56+ cells after purification of CD34+ cells from UCB. [file Image_1.tif]

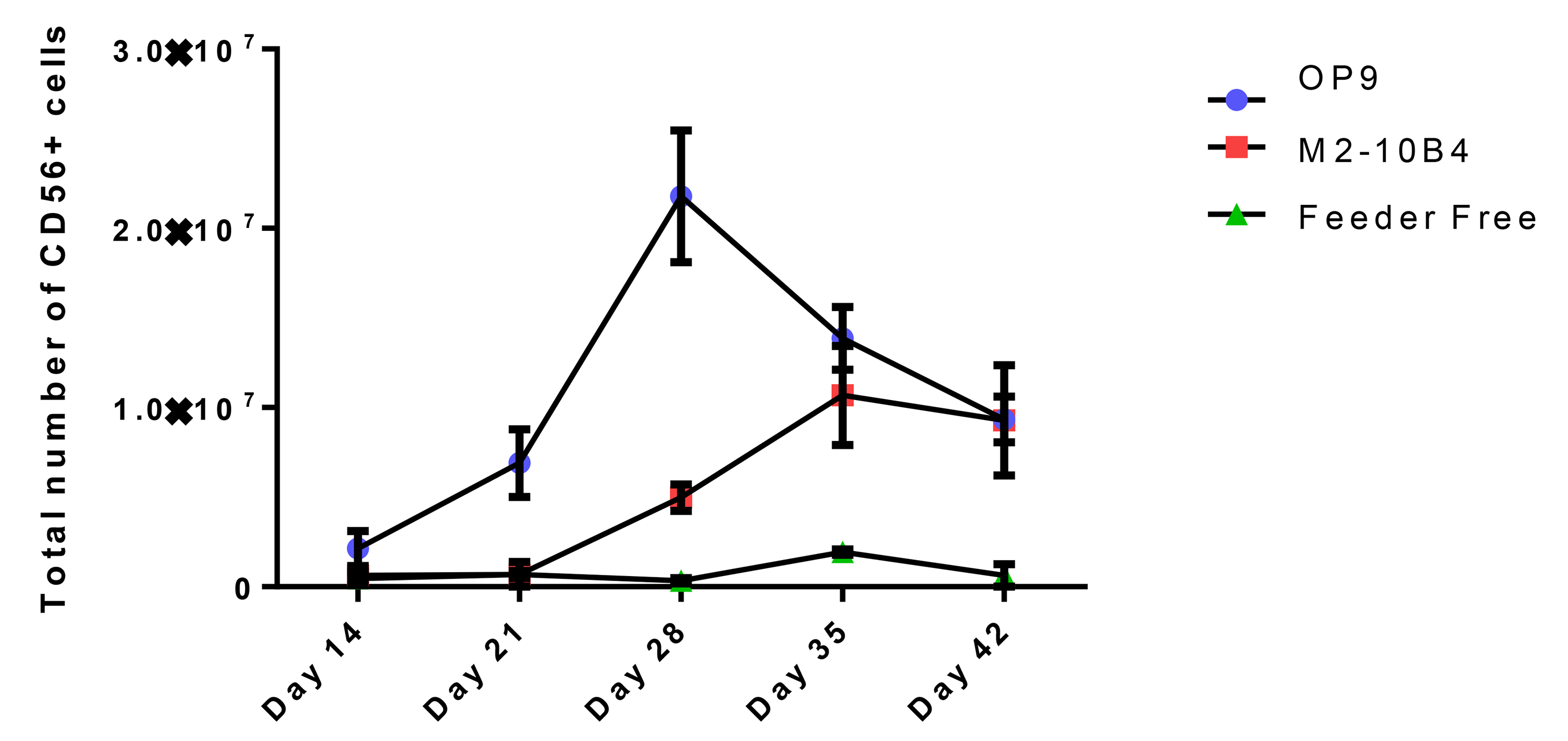

Supplement: Figure S2 — Total number of CD56+ cells at different time points during the in vitro differentiation protocol with three different culture conditions: OP9 and M2-10B4 cells coculture (N:4) and feeder-free (N:2). [file Image_2.tif]

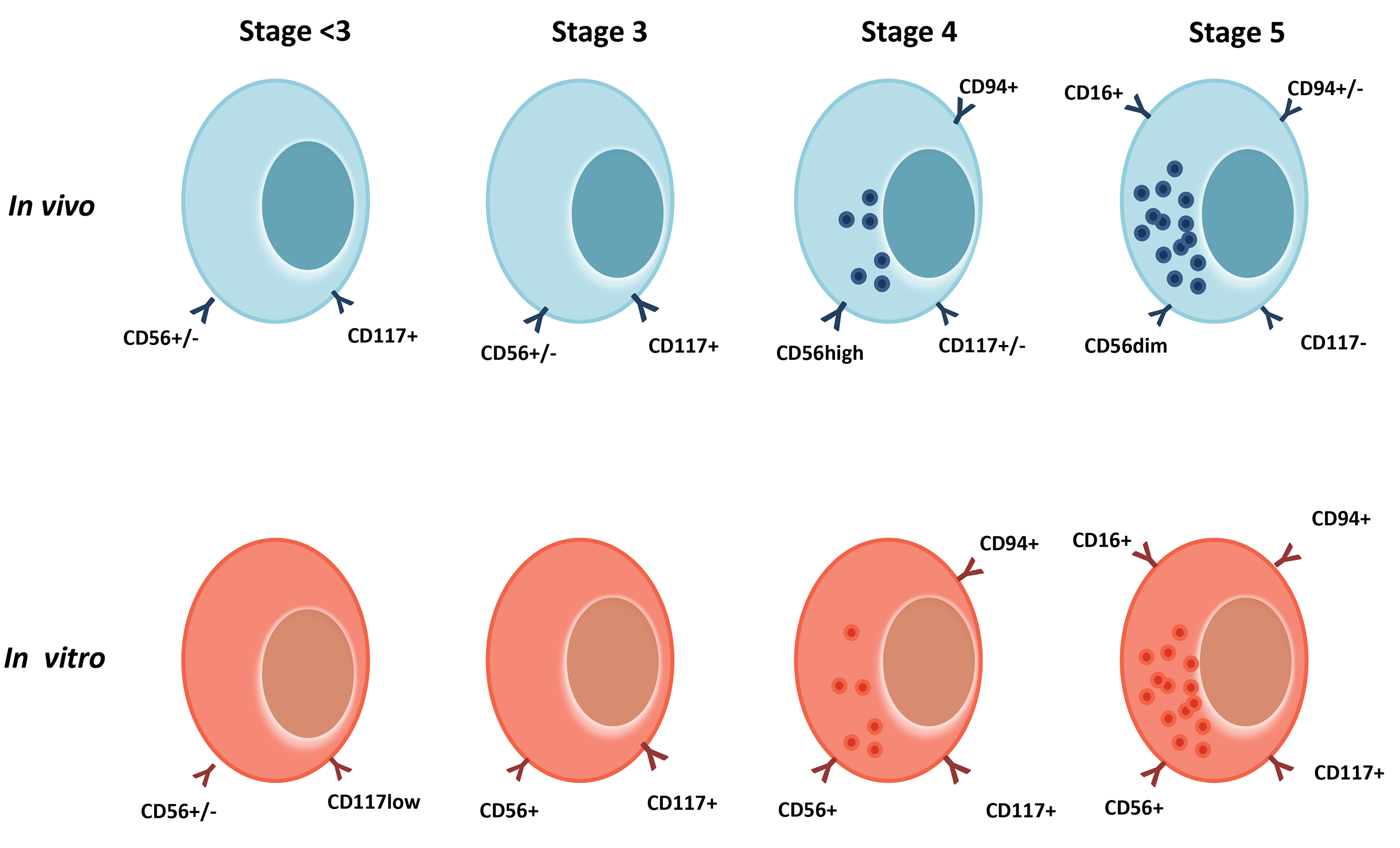

Supplement: Figure S3 — Key markers expressed at different stages through natural killer cell differentiation/maturation in vivo and in vitro. [file Image_3.tif]

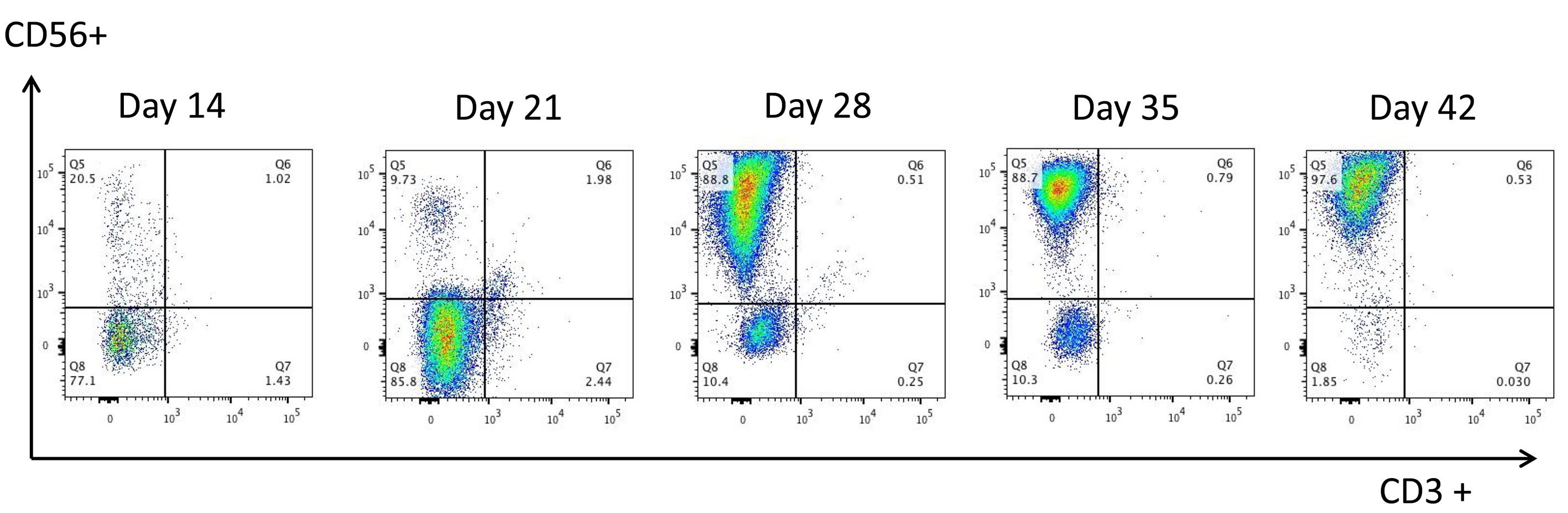

Supplement: Figure S4 — Dot-plots of the expression of CD3 at different stages of natural killer cells during the differentiation protocol. [file Image_4.tif]
